# Modeling stream fish distributions using interval‐censored detection times

**DOI:** 10.1002/ece3.2295

**Published:** 2016-07-13

**Authors:** Mário Ferreira, Ana Filipa Filipe, David C. Bardos, Maria Filomena Magalhães, Pedro Beja

**Affiliations:** ^1^ EDP Biodiversity ChairCIBIO/InBIOCentro de Investigação em Biodiversidade e Recursos Genéticos da Universidade do PortoCampus Agrário de Vairão, R. Padre Armando Quintas4485‐661VairãoPortugal; ^2^ CEABN/InBIOCentro de Ecologia Aplicada “Professor Baeta Neves”Instituto Superior de AgronomiaUniversidade de LisboaTapada da Ajuda1349‐017LisboaPortugal; ^3^ School of PhysicsThe University of MelbourneParkvilleVictoria3010Australia; ^4^ Departamento de Biologia Animal, Faculdade de Ciências de LisboacE3c, Centro de Ecologia, Evolução e Alterações AmbientaisCampo Grande, Bloco C21749‐016LisboaPortugal

**Keywords:** Distribution modeling, hierarchical Bayesian models, imperfect detection, occupancy‐detection modeling, stream fish, survival analysis, time to first detection

## Abstract

Controlling for imperfect detection is important for developing species distribution models (SDMs). Occupancy‐detection models based on the time needed to detect a species can be used to address this problem, but this is hindered when times to detection are not known precisely. Here, we extend the time‐to‐detection model to deal with detections recorded in time intervals and illustrate the method using a case study on stream fish distribution modeling. We collected electrofishing samples of six fish species across a Mediterranean watershed in Northeast Portugal. Based on a Bayesian hierarchical framework, we modeled the probability of water presence in stream channels, and the probability of species occupancy conditional on water presence, in relation to environmental and spatial variables. We also modeled time‐to‐first detection conditional on occupancy in relation to local factors, using modified interval‐censored exponential survival models. Posterior distributions of occupancy probabilities derived from the models were used to produce species distribution maps. Simulations indicated that the modified time‐to‐detection model provided unbiased parameter estimates despite interval‐censoring. There was a tendency for spatial variation in detection rates to be primarily influenced by depth and, to a lesser extent, stream width. Species occupancies were consistently affected by stream order, elevation, and annual precipitation. Bayesian *P*‐values and AUCs indicated that all models had adequate fit and high discrimination ability, respectively. Mapping of predicted occupancy probabilities showed widespread distribution by most species, but uncertainty was generally higher in tributaries and upper reaches. The interval‐censored time‐to‐detection model provides a practical solution to model occupancy‐detection when detections are recorded in time intervals. This modeling framework is useful for developing SDMs while controlling for variation in detection rates, as it uses simple data that can be readily collected by field ecologists.

## Introduction

Species distribution models (SDMs) are widely used for research on biodiversity patterns and processes, and for informing conservation action and wildlife management (Guisan and Thuiller 2005). Despite their value, SDMs may often be biased due to the use of datasets including false absences (Lobo et al. 2010; Kéry 2011; Dorazio 2012; Lahoz‐Monfort et al. 2014) because failure to detect a species where it is present is a common source of error in biological surveys (Guillera‐Arroita et al. 2014; and references therein). This problem may be solved using occupancy‐detection modeling, whereby presence–absence and detectability given presence are jointly modeled in relation to covariates (MacKenzie et al. 2006), although only recently this approach has been considered in SDM development (Chen et al. 2013; Kéry et al. 2013; Lahoz‐Monfort et al. 2014).

Occupancy‐detection modeling is generally based on data from replicate discrete surveys conducted at, at least, a subset of sampling units (sites; MacKenzie et al. 2006). Replicated surveys may be made by visiting sites more than once, but they may also be conducted at the same site on a single visit but partitioned by time, observer or method, or they can be conducted at different locations within a site (MacKenzie et al. 2006; Guillera‐Arroita 2011). In the removal design (MacKenzie et al. 2006), surveying is halted at a site once the species is detected and it was proposed that detection probabilities could be modeled as functions of covariates that vary across sites and also those (“such as local environmental conditions, time of day, or survey or experience”) that vary across surveys. This removal design is therefore a very general approach to modeling first‐detections where survey effort is treated as a series of discrete surveys. As such a crucial issue is exactly how the detection probabilities are modeled parametrically; MacKenzie et al. (2006) suggested a logistic model using a combination of covariates that varied only between sites and those that varied between both sites and surveys.

A potentially more natural approach for developing SDMs while controlling for imperfect detection is to model the observation process as continuous process (e.g., a Poisson point process in time) and use the time needed to first detect a species, rather than a detection/nondetection history (Garrard et al. 2008; Guillera‐Arroita et al. 2011). Time to first detection is a decreasing function of detectability and is known to be affected by factors such as species abundance, species traits, and sampling efficiency (Garrard et al. 2013; McCarthy et al. 2013; Bornand et al. 2014). The method is based on survival analysis (Kleinbaum and Klein 2012), using distributions of times to first detection to parameterize a survival curve S(*t*) (i.e., the probability of a species remaining undetected before a given time *t*), and to separate the probability of occupancy from the probability of detection given occupancy. The method has been mostly used in visual surveys of vascular plants (e.g., Garrard et al. 2008, 2013; McCarthy et al. 2013; Bornand et al. 2014), but it is likely useful for a wide range of taxonomic groups and sampling methods.

One problem of time‐to‐detection approaches is that the exact time when a species was first detected may be difficult to estimate precisely in some circumstances due, for instance, to sampling or recording constraints. In case of bird point counts, it is common practice to divide the count in time intervals, and recording species detections in each interval rather than at specific points in time (e.g., Alldredge et al. 2007). Likewise, in surveys of aquatic organisms using for instance nets, electrofishing or traps, species detections can often be recorded only within time intervals, and so the exact time to first detection is not known precisely (e.g., Beja and Alcazar 2003). In conventional survival analysis, this problem has been described as interval‐censoring, commonly resulting when periodic assessments (e.g., clinical or laboratory examinations) are used to assess if an event of interest has occurred (Radke 2003; Chen et al. 2012; Kleinbaum and Klein 2012). In these circumstances, the event is known only to have occurred before a given assessment (right‐censoring) and after the previous assessment (left‐censoring), corresponding to the upper and lower bounds of a time interval. Common approximations for dealing with interval‐censored data assume exact times (e.g., events occurring at the lower‐bound, midpoint or upper bound of the interval); these approaches are arbitrary and can result in biased estimates of the survival curve and the effects of covariates (Radke 2003; Chen et al. 2012). We therefore avoid such approximations in applying interval‐censored survival analysis to occupancy‐detection modeling.

In this study, we developed a time‐to‐first‐detection framework in the context of SDMs, using a modified formulation of interval‐censored survival analysis to deal with detections recorded in time intervals (Kleinbaum and Klein 2012). This provides a natural and coherent parameterization of detection probabilities for the removal design (MacKenzie et al. 2006) as a function of site‐covariates and arbitrary time intervals. This parameterization is achieved by way of a detection rate that may be constant (exponential survival model) or vary with elapsed time (e.g., the 2‐parameter Weibull survival model), and can be modeled as a function of site‐covariates using a log‐linear model.

The approach is illustrated using stream fish distributions, for which detection may greatly vary across sampling sites, and times to detection are difficult to estimate precisely due to sampling constraints (Zalewsky and Cowx 1990; Reynolds 1996; Penczak and Głowacki 2008). In detail, we examined if the interval‐censored time‐to‐detection approach allows building reliable models when imperfect detection is a potential drawback. We then used these models to extrapolate distributions of fish throughout the catchment streams. Finally, we discuss potential applications of the interval‐censored time‐to‐detection model to different datasets that may often be collected by field ecologists.

## Methods

### Fish and environmental data

Descriptions of the study area, and of methodological details for species surveys and, the collection of environmental data are provided in Appendix S1. We studied time‐to‐detection data for freshwater fish species sampled using electrofishing (Reynolds 1996), in 50‐m reaches (hereafter sites) distributed across the river Sabor catchment (NE Portugal). Sampling was conducted in the summer of 2012 at 89 sites, while no conditions for fish occurrence due to lack of surface water were recorded at another 95 sites. The study focused on the six most prevalent species (>20 sampling sites), including four natives (*Luciobarbus bocagei*,* Pseudochodrostoma duriense*,* Squalius alburnoides*, and *Squalius carolitertti*) and two exotics (*Gobio lozanoi* and *Lepomis gibbosus*). At each site, we carried out an electrofishing session lasting for 15–25 min, with longer surveys used in wider streams to cover adequately the entire sector. The first detection of each species was recorded in 5‐min intervals due to practical constraints associated with electrofishing sampling.

Detection probabilities were modeled in relation to stream width and depth, because these variables strongly affect detectability by inducing variations in electrofishing efficiency (Reynolds 1996) and in fish abundances (MacKenzie et al. 2006; McCarthy et al. 2013). Occupancy probabilities were modeled in relation to annual precipitation, elevation, and Strahler's stream order, because these variables are known to strongly influence the distribution of stream fish in Mediterranean regions (Magalhães et al. 2002; Filipe et al. 2004; Ferreira et al. 2007), and they could be readily used to project the distribution models for the entire watershed.

### Neighborhood effects

Modeling included neighborhood effects to account for potential biases resulting from spatial autocorrelation of the data, that is, lack of independence between the values of variables sampled at nearby locations (Legendre 1993). We employed autologistic models (Besag 1974; Augustin et al. 1996; Gumpertz et al. 1997; Hoeting et al. 2000; Bardos et al. 2015) for species occurrence and surface water presence; *W*_*i*_ = 1 denotes water presence at site *i*, while *Z*_*i*_ = 1 indicates true species presence. These models include an autocovariate that models the distance‐weighted influence on response variables of surrounding response values, and a corresponding parameter allowing estimation of the strength of neighborhood effects. The autocovariate was constructed as a weighted sum over neighborhood responses, not as a weighted mean, following the work of Bardos et al. (2015). We used an inverse‐distance weighting, based on hydrological distance (stream length) in km, with a long‐distance cut off of 30 km (above which the weighting is zero) and a short‐distance cut off of 5 km, below which the weighting remains at 1/5, encoding the idea that the influence of particularly close sites does not increase without limit. The auto covariates at site i are therefore:

(1) $$\begin{array}{cc} & Wsp_{i}=\sum_{\begin{array}{c} k\neq i\\ k\leq N_{s}\\ d_{ik}\leq 30 \end{array}}\min\left({\frac{1}{d}}_{ik},\frac{1}{5}\right)W_{k}\\ & Zsp_{i}=\sum_{\begin{array}{c} k\neq i\\ k\leq N_{s}\\ d_{ik}\leq 30 \end{array}}\min\left({\frac{1}{d}}_{ik},\frac{1}{5}\right)Z_{k} \end{array}$$

where $N_{s}\;=\;184$ is the total number of sampling sites (including ‘dry’ sites) and $d_{ik}$ is the hydrological distance in km between sites i and k. Different long‐distance cut offs were tested but the 30‐km limit was retained because each site had at least two other sampling sites in its 30‐km neighborhood, and because it efficiently removed autocorrelation in model residuals as judged through Moran's I correlograms (Legendre and Legendre 2012).

### Species distribution models

We use WinBUGS to estimate the autologistic models for water availability and true species presence–absence; $\chi_{i}=\mathrm{Pr}\left(W_{i}=1|W_{-i}\right)$ denotes the conditional probability of water presence at site *i*, given water presence–absence at all other sites (denoted $W_{-i}$) and similarly $\psi_{i}=\mathrm{Pr}\left(Z_{i}=1|Z_{-i}\right)$ is the conditional probability of true occurrence at site i. $Z_{i}$ depends on *W*_*i*_ and each depends on a common set *X*_*ji*_ *j *=* *1,2,…, *n* of covariates, via autologistic models

(2) $$\begin{array}{cc} \text{logit}\left(\chi_{i}\right) & =\alpha_{0}+\alpha_{1}X_{1i}+\cdots +\alpha_{n}X_{ni}+\alpha_{\mathrm{auto}}Wsp_{i}\\ \text{logit}\left(\psi_{i}\right) & =\beta_{0}+\beta_{1}X_{1i}+\cdots +\beta_{n}X_{ni}+B\left(W_{i}-1\right)+\beta_{\mathrm{auto}}Zsp_{i} \end{array}$$

where $\alpha_{\mathrm{auto}},\alpha_{0},\alpha_{1},\ldots$ and $\beta_{\mathrm{auto}},\beta_{0},\beta_{1},\ldots$ are regression coefficients and B is a large positive constant (e.g., $10^{9}$) that ensures the probability of presence $\psi_{i}$ is effectively zero when water is absent (*W*_*i*_ = 0).

We related true occupation to observed species presence and detection times via a model based on interval‐censored exponential survival models (Chen et al. 2012; Kleinbaum and Klein 2012). Under interval‐censoring (see Appendix S2), the likelihood of detecting a species at each sampling site, in the time interval $\left(t_{1,i},t_{2,i}\right]$, during a survey of duration *T*_*i*_, is given in terms of parametric detection‐time distributions *S*(*t*) = *S*(*t*,* θ*):

(3) $$\begin{array}{cc} & l\left(\delta_{i}=1,t_{1,i},t_{2,i}|\theta_{i},\psi_{i}\right)=\psi_{i}\left(S\left(t_{1,i},\theta_{i}\right)-S\left(t_{2,i},\theta_{i}\right)\right)\\ & l\left(\delta_{i}=0|T_{i},\theta_{i},\psi_{i}\right)=\psi_{i}S\left(T_{i},\theta_{i}\right)+\left(1-\psi_{i}\right) \end{array}$$

for $i\in \{1,2,\ldots N_{s}\}$, where *δ*_*i*_ is an indicator variable specifying whether the species was detected (1) or not (0) at site *i*, $\theta_{i}$ is a vector of detection‐time distribution parameters at site *i*,* t*1,i and *t*2,i are the lower and upper bounds of the time interval in which the species was detected at site *i*,* T*_*i*_ is the total survey time.

For the analysis here, we use the exponential detection‐time distribution $S\left(t\right)=e^{-\lambda t}$, where the detection rate λ is the sole parameter, so that the likelihood is then

(4) $$\begin{array}{cc} & l\left(\delta_{i}=1,t_{1,t},t_{2,t}|\lambda_{i},\psi_{i}\right)=\psi_{i}\left(e^{-\lambda_{i}t_{1,i}}-e^{-\lambda_{i}t_{2,i}}\right)\\ & l\left(\delta_{i}=0|\lambda_{i},\psi_{i},T_{i}\right)=\psi_{i}\left(e^{-\lambda_{i}T_{i}}\right)+\left(1-\psi_{i}\right) \end{array}$$

and we use a log‐linear model for the detection rate *λ*_*i*_ at site *i*

(5) $$\log\left(\lambda_{i}\right)=\gamma_{0}+\gamma_{1}Y_{1i}+\cdots +\gamma_{m}Y_{mi}$$

where *Y*_*ji*_, *j *=* *1,2,…, *m*, comprise linear and quadratic terms for environmental covariates and $\gamma_{0},\gamma_{1}$, … are regression coefficients.

### Simulations for the detectability model

We conducted simulations to evaluate the performance of the interval‐censored exponential model for detection data resulting from a study design comparable to ours, using an approach similar to Garrard (2009). For a set of *K *=* *150 sampling sites, we used a Bernoulli trial with a probability ψ to generate the “known” occupancy status at each site. Detection times given occupancy were generated using a random generator of exponential distribution times, with detection rate *λ*. We set a maximum time for sampling at each site of *T*_max_ = 15 min, with nondetections occurring when sites were vacant or when time to detection exceeded *T*_max_. Simulations were performed considering nine combinations of parameters, with occupancy set to ψ = 0.25, 0.5 and 0.75, and the detection rate set to λ = 0.20, 0.10 and 0.07. These detections rates correspond to mean detection times of 5, 10, and 15 min, respectively. For each combination of parameters, we ran 1000 times.

### Model building and evaluation

To avoid model instability and allow comparisons between parameters, all environmental covariates were standardized to zero mean and unit standard deviation. The detection component was fit to the full model, including second order polynomials of both depth and width, thereby allowing for nonlinear changes in detection in relation to covariates. The occupancy and water presence components were also fit to the full model, including the three large‐scale environmental variables and the neighborhood effects. We fitted full models instead of seeking more parsimonious models because there is at present considerable uncertainty on the most reliable methods to undertake selection in Bayesian models (e.g. Kéry 2010), the number of variables was low relative to sample sizes, and modeling was based on a small set of variables described in the literature to affect stream fish detection and occupancy. The effects of variables were judged from the 95% credible intervals, assuming that evidence for an effect is ambiguous when the credible interval of a parameter estimate includes zero (Kéry 2010).

Overall model fit was assessed using posterior predictive checks based on standard Bayesian *P*‐values (Gelman et al. 1996), measuring the discrepancy between observed and predicted detections at sampling sites. Extreme *P‐*values (e.g., >0.95 or <0.05) are indicative of poor fit, whereas values near 0.5 indicate well‐fitting models. Model discrimination ability was evaluated using an elaboration of the area under the receiver operating characteristic curve (AUC) in which posterior AUC distributions are calculated (Zipkin et al. 2012). Predicted probabilities of species presence cannot be directly compared to observed presences/absences, because false absences may occur (Garrard et al. 2013). In our study, AUC was based on comparisons between predicted detection probabilities and actual detections/nondetections at sites that were sampled (i.e., sites that were not dry), thereby providing an evaluation of the time‐to‐detection model fit. Probability of detecting a species at each site *i*, conditional on the sampling duration, *T*_*i*_, was based on the second part of eq. (4), as follows:

(6) $$\mathrm{Pr}\left(t_{i}<T_{i}|\psi_{i},\lambda_{i}\right)=\psi_{i}\left(1-e^{-\lambda_{i}T_{i}}\right)$$

This unconditional probability of detection integrates both the probability of the species being present at the site, and the conditional probability of detection given presence. We performed a fivefold cross‐validation, in order to obtain a true predictive performance measure (Broms et al. 2016): (1) we randomly divided the data in five sets; (2) withholding one set, we fitted the model to the remaining sets; (3) computed AUC for the withheld set; and (4) we repeated the process for every subset.

We used all draws of the estimates of eq. (5) to estimate posterior distributions and credible intervals of AUC values (ranging 0–1, where values >0.5 indicate progressively better discrimination ability) using the R package ROCR (Sing et al. 2005).

The posterior probabilities of species detection were also used against actual detections/nondetections to estimate spatial autocorrelation in model residuals. For each model, we constructed a Moran's I correlogram using the mean values of the residuals posterior distributions and evaluated the significance of Moran's I coefficients with Monte Carlo permutation tests using the R package APE (Paradis et al. 2004). To build the correlogram, pair wise distances were divided in classes such that a similar number of pairs was assigned to each class, thereby assuring comparable power in tests of significance across all distance classes (Legendre and Legendre 2012).

### Species distribution mapping

We developed occupancy probability maps comprising (1) posterior autologistic occupancy probabilities ${\overset{¯}{\psi }}_{i}$ for sampled sites $i\leq N_{s}$; and (2) extrapolated probabilities ${\overset{¯}{\psi }}_{i}$ for a further 1861 unsampled sites (with labels $i>N_{s}$) across the stream network of the Sabor catchment, for which neighborhood effects are extrapolated by treating sampled sites as though they are neighbors of each unsampled site, that is by applying eq. (1) to sites $i>N_{s}$. In case where eq. (1) reduces to a logistic model (i.e., $\alpha_{\mathrm{auto}}=\beta_{\mathrm{auto}}=0$), then for each extrapolation site $i>N_{s}$, ${\overset{¯}{\psi }}_{i}$ reduces to a posterior logistic occupancy probability. We used this extrapolation approach for neighborhood effects because including the unsampled sites as missing data in the autologistic model was computationally impractical in WinBUGS.

For computational convenience, the stream network was segmented according to the following criteria: (1) each first order stream was one segment; (2) one segment in higher order streams was the reach between two successive tributaries; and (3) long reaches were divided so that all segments were <1000 m. Each segment was then assigned with the environmental characteristics of the corresponding centroid. At each segment, we thus assumed that environmental conditions and neighborhood effects were constant, and there was no variation in the probabilities of water presence and species occupancy at 50‐m stream reaches. We used the mean estimated probabilities of species occupancy, and the standard deviation of the posterior distribution to produce the maps of predicted species distribution, and the uncertainty of model predictions. All spatial analysis and data manipulation were performed in ArcMap 10.0 (ESRI 2011).

### Model fit

Models were fit in WinBUGS (Lunn et al. 2000), by calling WinBUGS through the package R2WinBUGS (Sturtz et al. 2005) in R (R Core Team 2015), and handling the results back in R. Following a sensitivity analysis (Cressie et al. 2009), prior distributions of parameters were specified as normal distributions with zero mean and variance 10, truncated to the domain (‐10,10). We ran five chains of 100,000 iterations after a burn in of 50,000, and thinned the chains by 20 resulting in 12,500 simulations for each parameter. Convergence was assessed with the R‐hat statistic, which examines the variance ratio of the MCMC algorithm within and between chains across iterations. WinBUGS code is provided in Appendix S3.

## Results

The simulation results (Table 1) showed that at sample sizes similar to ours the interval‐censored model performed well. The simulated parameters were always well within the estimated credible intervals, and they were generally very close to the median parameter estimates. However, the occupation probability tended to be overestimated for lower levels of occupancy especially for lower detection rates.

**Table 1:** Performance of the interval‐censored time‐to‐detection model in retrieving parameter from simulated data. The simulated data were generated using nine combinations of parameters, including three levels each of occupancy probability (*Ψ*) and detection rate (*λ*). For each simulated condition, we present the median and credible intervals (in brackets) of parameter estimates based on the medians from 1000 simulations

Simulated parameters		Estimated parameters	
ψ	λ	$\hat{\psi }$	$\hat{\lambda }$
0.25	0.20	0.26 (0.15–0.36)	0.19 (0.09–0.33)
	0.10	0.28 (0.17–0.60)	0.09 (0.02–0.20)
	0.07	0.35 (0.16–0.63)	0.04 (0.01–0.14)
0.50	0.20	0.49 (0.39–0.60)	0.20 (0.15–0.27)
	0.10	0.48 (0.34–0.77)	0.10 (0.05–0.19)
	0.07	0.50 (0.32–0.73)	0.07 (0.03–0.15)
0.75	0.20	0.74 (0.65–0.83)	0.20 (0.16–0.26)
	0.10	0.72 (0.60–0.86)	0.11 (0.07–0.15)
	0.07	0.69 (0.52–0.86)	0.07 (0.05–0.13)

The occupation‐detection models for the six species showed adequate convergence of parameter estimates as judged from the R‐hat statistics. Bayesian *P‐*values were far from zero and one, ranging from 0.43 (*L. gibbosus*) to 0.64 (*S. alburnoides*), and thus model fit was considered adequate. Median AUCs estimated through cross‐validation ranged between 0.67 and 0.93 indicating that the discrimination ability between detection and nondetection sites was particularly high (AUC > 0.80) for all species but *L. gibbosus* (Table 2). Moran's I correlograms indicated that there was no significant autocorrelation in the residuals of species occupancy‐detection models.

**Table 2:** Mean parameter estimates and the corresponding 95% credible intervals (in brackets) of the best‐supported models used in the distribution mapping of six freshwater fish species. Values are shown for each level of the hierarchical model: water availability – probability of a site having water; occupancy – probability of species occupying a site; detection – detection rate of the species in sites where it is present. AUC is the area under the curve of the receiver operating characteristic. Highlighted in bold are parameters (except the intercept) with credible intervals excluding zero

Parameters	*L. bocagei*	*P. duriense*	*S. alburnoides*	*S. carolitertii*	*G. lozanoi*	*L. gibbosus*
Water availability						
Intercept	−0.44 (−1.69; 0.80)	−0.44 (−1.68; 0.81)	−0.43 (−1.67; 0.82)	−0.44 (−1.69; 0.82)	−0.43 (−1.68; 0.84)	−0.42 (−1.68; 0.82)
Elevation	0.37 (−0.40; 1.14)	0.37 (−0.40; 1.16)	0.37 (−0.40; 1.16)	0.37 (−0.41; 1.14)	0.37 (−0.40; 1.14)	0.37 (−0.40; 1.14)
Stream order	**2.55 (1.86; 3.35)**	**2.55 (1.86; 3.35)**	**2.55 (1.86; 3.35)**	**2.55 (1.86; 3.35)**	**2.55 (1.86; 3.35)**	**2.55 (1.86; 3.35)**
Precipitation	0.37 (−0.13; 0.90)	0.38 (−0.12; 0.89)	0.38 (−0.12; 0.89)	0.38 (−0.13; 0.89)	0.38 (−0.12; 0.91)	0.37 (−0.13; 0.89)
Neighborhood	−0.11 (−1.36; 1.12)	−0.10 (−1.36; 1.13)	−0.11 (−1.39; 1.09)	−0.11 (−1.38; 1.12)	−0.11 (−1.37; 1.12)	−0.12 (−1.35; 1.11)
Occupancy						
Intercept	−3.34 (−6.33; −0.72)	−0.87 (−3.95; 4.91)	1.40 (−1.39; 5.72)	−2.77 (−4.46; −1.21)	−4.93 (−8.19; −2.25)	−1.71 (−3.99; 1.67)
Elevation	−0.39 (−2.59; 1.02)	0.49 (−4.76; 2.50)	**3.06 (**−**0.05; 5.83)**	1.55 (0.78; 2.44)	−**5.25 (**−**8.39;** −**2.56)**	−**0.96 (**−**5.33; 1.61)**
Stream order	**3.79 (1.83; 6.93)**	2.18 (−0.88; 6.07)	−1.08 (−3.53; 0.93)	**1.34 (0.48; 2.35)**	1.72 (0.18; 3.70)	−0.96 (−3.06; 1.15)
Precipitation	−**1.89 (**−**4.02;** −**0.36)**	−1.84 (−7.09; 0.38)	−**4.02 (**−**7.46;** −**0.58)**	−**1.03 (**−**2.24;** −**0.13)**	−1.23 (−3.44; 0.87)	−1.11 (−3.5; 0.56)
Neighborhood	−0.34 (−4.11; 3.65)	1.97 (−2.37; 7.43)	0.25 (−4.91; 4.97)	**3.06 (0.54; 5.30)**	−1.12 (−4.75; 2.42)	3.66 (−2.49; 7.46)
Detection						
Intercept	−1.15 (−2.03; −0.19)	−2.47 (−3.05; −1.14)	−3.06 (−3.81; −2.13)	−1.00 (−1.51; −0.54)	−1.54 (−2.05; −1.08)	−3.17 (−3.86; −2.18)
Width	−1.03 (−4.00; 1.82)	0.56 (−2.57; 2.27)	**4.01 (1.28; 6.37)**	−0.53 (−2.29; 1.11)	−0.13 (−2.05; 1.64)	0.55 (−2.06; 2.44)
Width^2^	2.06 (−0.79; 5.85)	−0.01 (−1.56; 2.60)	−**5.40 (**−**8.43;** −**2.11)**	0.36 (−1.03; 1.96)	0.31 (−1.22; 2.16)	0.45 (−1.27; 3.05)
Depth	**2.84 (0.18; 5.25)**	**1.80 (0.46; 3.25)**	0.46 (−1.63; 2.16)	1.42 (−0.19; 3.12)	0.41 (−1.81; 2.64)	0.15 (−2.21; 2.34)
Depth^2^	−**3.01 (**−**5.33;** −**0.42)**	−**1.59 (**−**2.82;** −**0.45)**	−0.30 (−1.88; 2.18)	−**1.49 (**−**3.02;** −**0.01)**	−0.57 (−2.93; 1.92)	0.32 (−1.94; 3.4)
AUC	0.92 (0.80; 1.00)	0.83 (0.53; 0.96)	0.83 (0.63; 0.94)	0.83 (0.61; 1.00)	0.93 (0.68; 1.00)	0.67 (0.14; 0.94)
Bayesian *P*‐value	0.54	0.58	0.64	0.55	0.50	0.43

**Figure 1:**
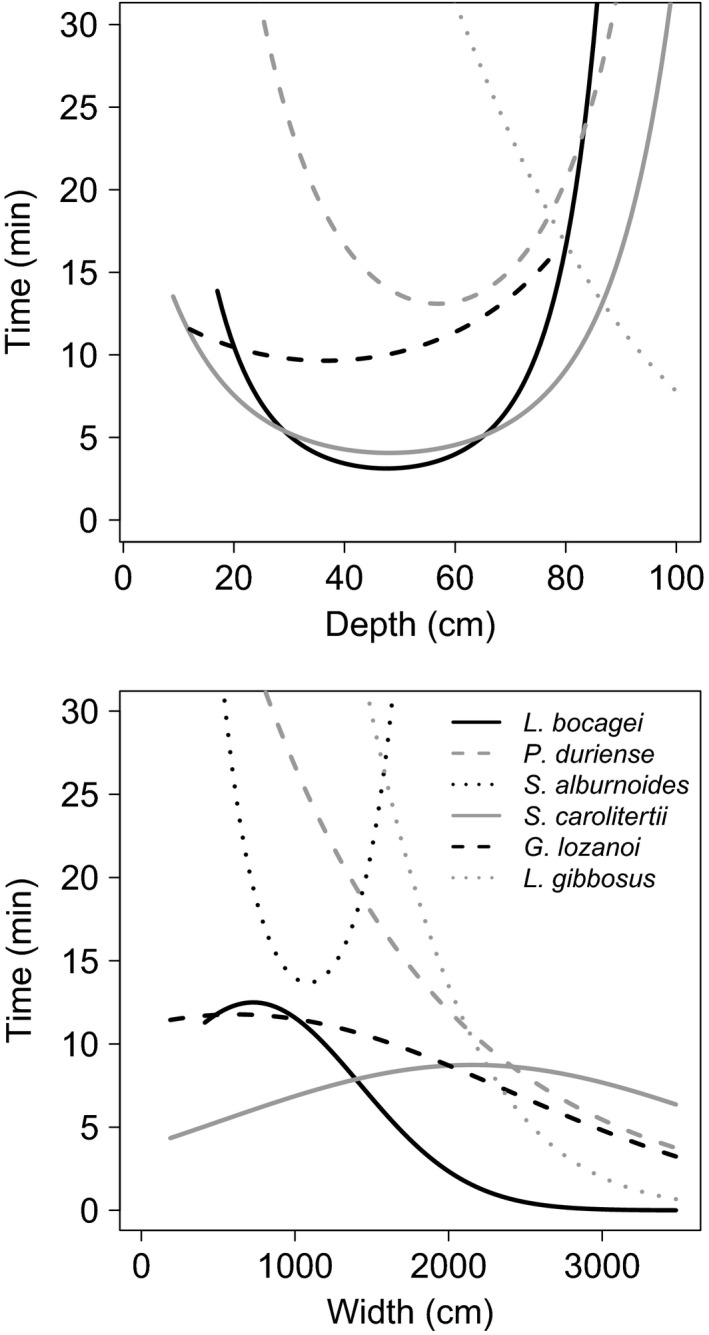
Variation in median times to first detection of each species with 0.9 success probability if species is present, as a function of stream depth and width. Curves were derived from the detection models in Table 2, by varying the values of one variable conditioning on the mean values of other covariates in the model.

There was evidence for depth influencing the detection probabilities of *L. bocagei*,* P. duriense*, and *S. carolitertii*, as the credible intervals of parameter estimates for the linear (except *S. carolitertii*) and quadratic terms did not overlap zero (Table 2). These results suggested a U‐shaped relationship with the median time to first detection, with shorter detection times when the stream was neither too shallow nor too deep (Fig. 1). In case of width, the credible intervals did not overlap zero in the model developed for *S. alburnoides*, suggesting also a U‐shaped relationship (Fig. 1). The probability of the stream channel having surface water during the sampling visit was positively related to stream order, elevation and precipitation, but the latter two effects were ambiguous because the credible intervals overlapped zero (Table 2, Appendix S4). The probability of occupancy in sites with surface water was positively related to stream order for *L. bocagei* and *S. carolitertii*; elevation had a positive effect on *S. alburnoides*, and a negative effect on *G. lozanoi* and *L. gibbosus;* and precipitation had a negative effect on *L. bocagei*,* S. alburnoides*, and *S. carolitertii* (Table 2, Appendix S4). Evidence for positive neighborhood effects was found for *S. carolitertii* (Table 2).

**Figure 2:**
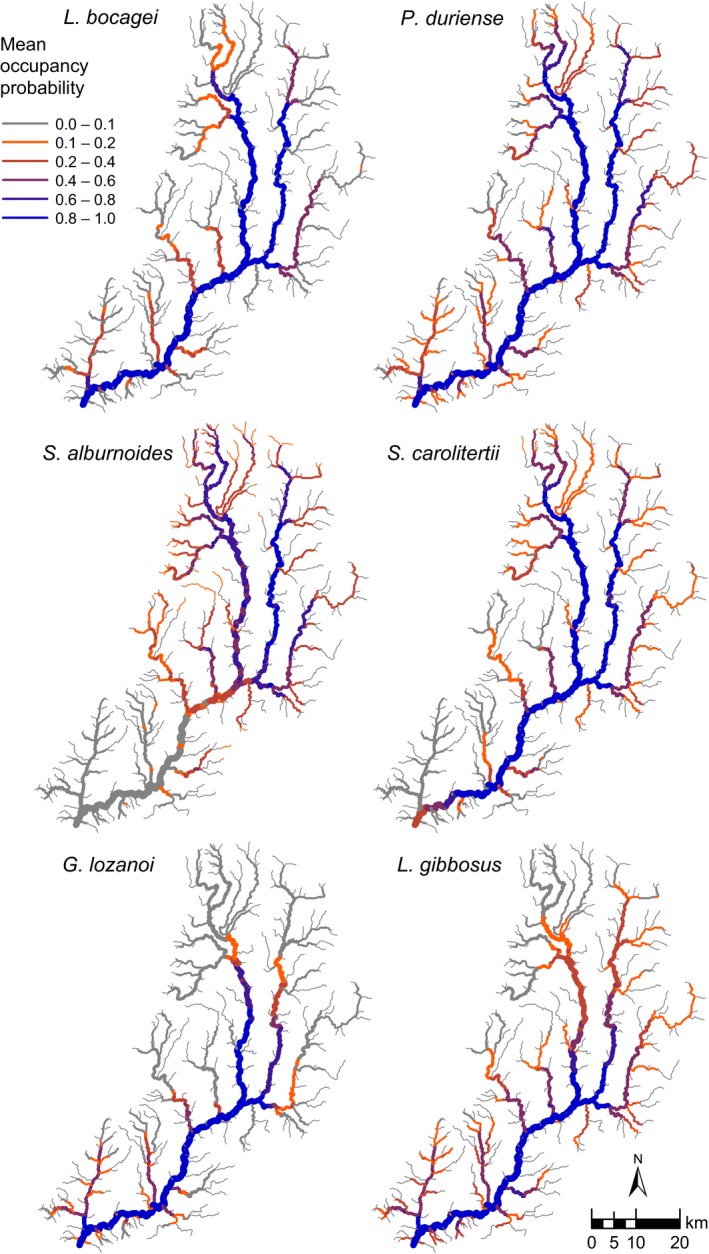
Predicted occupancy probabilities of six fish species across the river Sabor catchment, combining the probabilities of surface water being present in the watercourse, and the conditional probabilities of occupancy given water presence. Line width is proportional to stream order.

Maps of predicted distribution indicated that *L. bocagei*,* P. duriense*, and *S. carolitertti* were widespread, occupying most of the main river and its two largest tributaries (Fig. 2). *S. alburnoides* was more restricted, occurring primarily in the upper reach of the Sabor and the two main tributaries. From the two exotic species, *G. lozanoi* occurred primarily in the downstream reaches of the main river and its largest tributary, whereas *L. gibbosus* was more widespread, although it was also absent from upstream reaches and smallest tributaries (Fig. 2). Uncertainty in model predictions was low to moderate, and it was highest for *P. duriense*,* L. gibbosus*, and *S. alburnoides* (Appendix S5). In most cases, uncertainty in species occupancy probability tended to be higher in the tributaries and in upper river reaches, where it was affected by uncertainties in whether the watercourses were dry or not.

## Discussion

Our work expanded the time‐to‐detection model (Garrard et al. 2008, 2013) to deal with detections collected in time intervals (interval‐censoring) and illustrated its value for modeling species distribution using stream fish as a case study. The environmental correlates of occupancy identified for each species were in line with previous research on Mediterranean stream fish (e.g., Magalhães et al. 2002; Filipe et al. 2004; Ferreira et al. 2007), suggesting that models successfully incorporated key factors influencing species distributions. For most species, we found significant spatial variation in detectability, supporting the importance to control for imperfect detection in distribution modeling studies (Guillera‐Arroita et al. 2014; Lahoz‐Monfort et al. 2014). Overall, our approach should provide a useful addition to the toolbox of field ecologists modeling species distributions while controlling for imperfect detection (Chen et al. 2013; Lahoz‐Monfort et al. 2014).

Our study was based on the exponential model, which has been used in time‐to‐detection studies (Garrard et al. 2008, 2013), and it was considered a convenient choice due to its simplicity and its wide applicability (Kleinbaum and Klein 2012). The exponential is the simplest of the parametric survival models (Kleinbaum and Klein 2012), where times to detection are described by only one parameter and detections are assumed to occur at a constant rate (Garrard et al. 2008). Due to its memoryless property (Murphy et al. 2002), time elapsed in previous intervals does not alter detection probability for a subsequent sampling interval, and thus the exponential distribution cannot model increases or decreases in detectability during each survey. This limitation may be overcome using other parametric survival models, but exploring these possibilities were beyond the scope of our study. In contrast to previous time‐to‐detection studies (Garrard et al. 2008, 2013), our study was based on detections recorded in 5‐min time intervals rather than continuously. This was unavoidable, because during electrofishing, it is nearly impossible to keep a continuous track of each species captured, due to logistic constraints and difficulties in species identification. Therefore, we have used a modification of the time‐to‐detection approach based on interval‐censored survival analysis (Chen et al. 2012; Kleinbaum and Klein 2012), because common approximations assuming for instance events occurring at the lower‐bound, midpoint or upper bound of the interval may result in biased estimates of the survival curve and the effects of covariates (Radke 2003). Simulations showed that our approach provides unbiased estimates of detection rates and occupancy probabilities, suggesting that the method performs well in retrieving simulated values under conditions similar to our sampling design. It should be noted, however, that the occupancy probability tended to be overestimates for rare species (values of 0.25 in prevalence).

In four of six species, we found that variation in detectability across sites was influenced by stream depth, stream width, or both, and that responses to these variables varied across species. These effects may reflect variation in electrofishing efficiency, which is generally expected to be lower when water is too deep or too shallow, and when rivers are very wide (e.g., Zalewsky and Cowx 1990; Penczak and Głowacki 2008). Electrofishing efficiency is known to be affected by factors such fish size, shape, and behavior (e.g., benthic versus pelagic) (e.g., Zalewsky and Cowx 1990; Penczak and Głowacki 2008), which may explain to at least some extent the differences observed across species. It is also possible that effects of width and depth were mediated by their strong influence on Mediterranean stream fish abundances (e.g., Ferreira et al. 2007), which in turn may have major effects on species detection probabilities (MacKenzie et al. 2006; McCarthy et al. 2013). Different species reach the highest abundances in stream sectors of different width and depth (e.g., Ferreira et al. 2007), which may also contribute to explain changes in detection across species. Whatever the reasons, the results obtained provide empirical support to the view that accounting for imperfect detection is important when undertaking species distribution modeling (Lahoz‐Monfort et al. 2014). This may be particularly relevant when focusing on aquatic species such as fish and amphibians, because organisms living underwater are notoriously difficult to sample and may be highly affected by imperfect detection (Głowacki 2011), thereby calling for the use of modeling techniques controlling for variation in detectability (Comte and Grenouillet 2013; Ferreira and Beja 2013).

Modeling results revealed relationships between occupancy probabilities and environmental variables that are in line with the results from other studies carried out in Mediterranean streams, highlighting in particular the strong effect of stream order on occupancy (Magalhães et al. 2002; Filipe et al. 2004; Ferreira et al. 2007). For instance, we found that occupancy by *L. bocagei* and *P. duriensis* strongly increased with stream order, which is in line with observations elsewhere showing that barbel and straight‐mouth nase to be more prevalent in higher order streams. Overall, results suggest that time‐to‐detection modeling was successful in identifying key factors affecting fish distribution, while controlling for variation in detectability. It is noteworthy, however, that this component of the hierarchical model accounted only for the probability of occupancy when there is water in the watercourse, because part of the streams were dry and thus unavailable for occupation by the target species. This was dealt with by modeling the probability of water presence in relation to environmental variables as an additional component of the hierarchical model, using binary draws from this probability to simulate surface water availability, then predicting the probability, given water availability, of fish occupancy of any 50‐m reach of the stream network. Results indicated that the probability of water presence was mainly related to stream order, with headwater streams of order one and two tending to be dry and thus without conditions for fish, while streams and rivers of order three and above had a high probability of having water. This pattern is common in Mediterranean streams and elsewhere, where headwaters dry and as the stream channel increases in size downstream, surface water remains in pools or in surface flowing (Lake 2003; Robson et al. 2013). We thus suggest that both the presence of water and the detection of species given water presence should be routinely considered when modeling the distribution of aquatic organisms along stream networks and in other waterbodies (e.g., pond breeding amphibians; Ferreira and Beja 2013), providing a more realistic account of two potentially distinct processes affecting occupancy.

Evaluation of model discrimination ability for occupancy‐detection models is difficult, because true absences are unknown, and so predicted probabilities of species occupancy cannot be directly compared with observed presences/absences (Garrard et al. 2013). To circumvent this problem, Garrard et al. (2013) evaluated occupancy‐detection models by comparing the observed and predicted proportion of sites where each species was detected. Here, we expanded this approach, using a variant of the AUC method described by Zipkin et al. (2012) to compare predicted detection probabilities with observed detections/nondetections, which avoided any assumptions about the characteristics of nondetections. AUC is a standard method for evaluating species distribution models (e.g., Kharouba et al. 2013) that provides a more complete characterization of model discrimination ability than the simple comparison of the observed and predicted proportion of species detections. In contrast to Zipkin et al. (2012) we used AUC to estimate the discrimination ability between detections and nondetections, and not between presences and absences.

The approach described here may find wide applicability where time‐to‐detection approaches are sought to control for imperfect detection in occupancy studies (e.g., Garrard et al. 2008, 2013), but where a species detection can only be determined to lie in an interval obtained from a sequence of sampling intervals. This may be generally the case in electrofishing studies such as ours, but the problem may also occur over a wide range of circumstances. For instance, sampling of aquatic organisms in shallow waters often involve dip‐netting during fixed time intervals (Beja and Alcazar 2003). Also, during bird counts it is common to register detections in time intervals (Alldredge et al. 2007), because it is impractical to register the exact moment when each individual was seen or heard. Finally, in studies involving periodic checking of traps (e.g., drift nets, mist nets, live traps for small mammals) it is possible to know that a capture event occurred after the trap was set but before it was checked, but the exact moment of capture it is often unknown. In all these cases, time‐to‐detection modeling may benefit from a wealth of methods developed to deal with interval‐censored data, which have been particularly well explored in the medical and veterinary sciences (e.g., Radke 2003; Chen et al. 2012). These methods allow extending the relatively simple case described in our study, by accommodating for instance variation in the duration of time intervals across sampling units, or by replacing the exponential by a more flexible model (e.g., Weibull) that can account for changes in detectability within each sampling occasion (e.g., Chen et al. 2012; Kleinbaum and Klein 2012). Overall, the interval‐censored time‐to‐detection model framework revealed as a promising approach for developing SDMs that could accommodate variation in detection rates, and we expect this approach to be tested in other case studies where time of first detection is not known precisely.

## Conflict of Interest

None declared.

## Supporting information


**Appendix S1.** Supplementary Methods.
**Appendix S2.** Interval‐censored time to detection model.
**Appendix S3.** Code used to fit the time to detection model using WinBUGS.
**Appendix S4.** Response curves to environmental variables.
**Appendix S5.** Maps of prediction uncertainty.
**Appendix S6.** Supplementary references.
