# Supplementary material for: Modeling stream fish distributions using interval‐censored detection times
Source: Ecol Evol. 2016 Jul 13;6(15):5530–41. doi: 10.1002/ece3.2295 (PMC4984523; doi:10.1002/ece3.2295)
Supplement: Supplementary file 1 — Appendix S1. Supplementary Methods. Appendix S2. Interval‐censored time to detection model. Appendix S3. Code used to fit the time to detection model using WinBUGS. Appendix S4. Response curves to environmental variables. Appendix S5. Maps of prediction uncertainty. Appendix S6. Supplementary references. [file ECE3-6-5530-s001.pdf]

Appendix S1 Supplementary Methods

Study area

The study was conducted in NE Portugal, in the river Sabor catchment (Figure S1), which covers a wide range of environmental conditions in terms of elevation (100-1500 meters above sea level), total annual precipitation (443 - 1163 mm), and mean annual temperature (6.9 – 15.6^{o}C). Climate is Mediterranean, with precipitation largely concentrated in October-March, while it is virtually absent in the hot summer months (June-August). Flow regime is highly seasonal, with most headwater streams drying out or being reduced to a series of disconnected pools in summer, though the main watercourse and some of the tributaries are permanent.

Fish sampling

Fish sampling was carried out at 50-m reaches of streams (hereafter referred to as sites) distributed across the watershed, during the summer of 2012 (June 13 to July 15, and August 28 to September 15). The reach length was selected based on previous studies showing that it is appropriate for describing spatial and temporal variations in Mediterranean stream fish assemblages (Magalhães et al., 2002, 2007). We visited 184 sites across the watershed, and sampled the 89 sites with flowing water or isolated pools. The remaining sites were totally dry, and so they could not sustain fish populations during the sampling period. The sites were selected in the field based on accessibility and representativeness, as long they were 4 to 10km apart from each other, and provided a thorough coverage of the watershed (Figure S1). Within each site, we sampled fish using a single anode electrofishing gear (350-750 V, 3–5A, DC), following standard procedures (Zalewsky & Cowx, 1990; Penczak & Głowacki, 2008). Electrofishing was always conducted by the same operator (M.F.), accompanied by a second operator to net fish displaced by electrofishing. Sampling was carried out during 15 to 25 minutes, with longer surveys used in wider streams to cover adequately the entire sector (Reynolds, 1996). During each sampling session, we electrofished progressively in the upstream direction, and transferred fish alive to containers distributed along the margins. Because most fish captured were small-sized or otherwise difficult to identify to species level without careful examination, identification and the estimate of times to first detection were made at the end of the sampling session. To this end, fish captured in each interval of 5 minutes were transferred to a different set of marked containers, which allowed the recording of species detections in 5-minute intervals. After identification fish were returned alive to the stream.

Sampling yielded 12 fish species (Table S1), of which four natives (*Luciobarbus bocagei*, *Pseudochodrostoma duriense*, *Squalius alburnoides*, and *Squalius carolitertti*) and two exotics (*Gobio lozanoi* and *Lepomis gibbosus*) were used in occupation-detection modelling. The remaining six species occurred too rarely (1 to 13 sites), and so they were discarded to reduce potential problems associated with a low number of events per variable in occupancy and detection models (e.g., Vittinghoff & McCulloch, 2007).

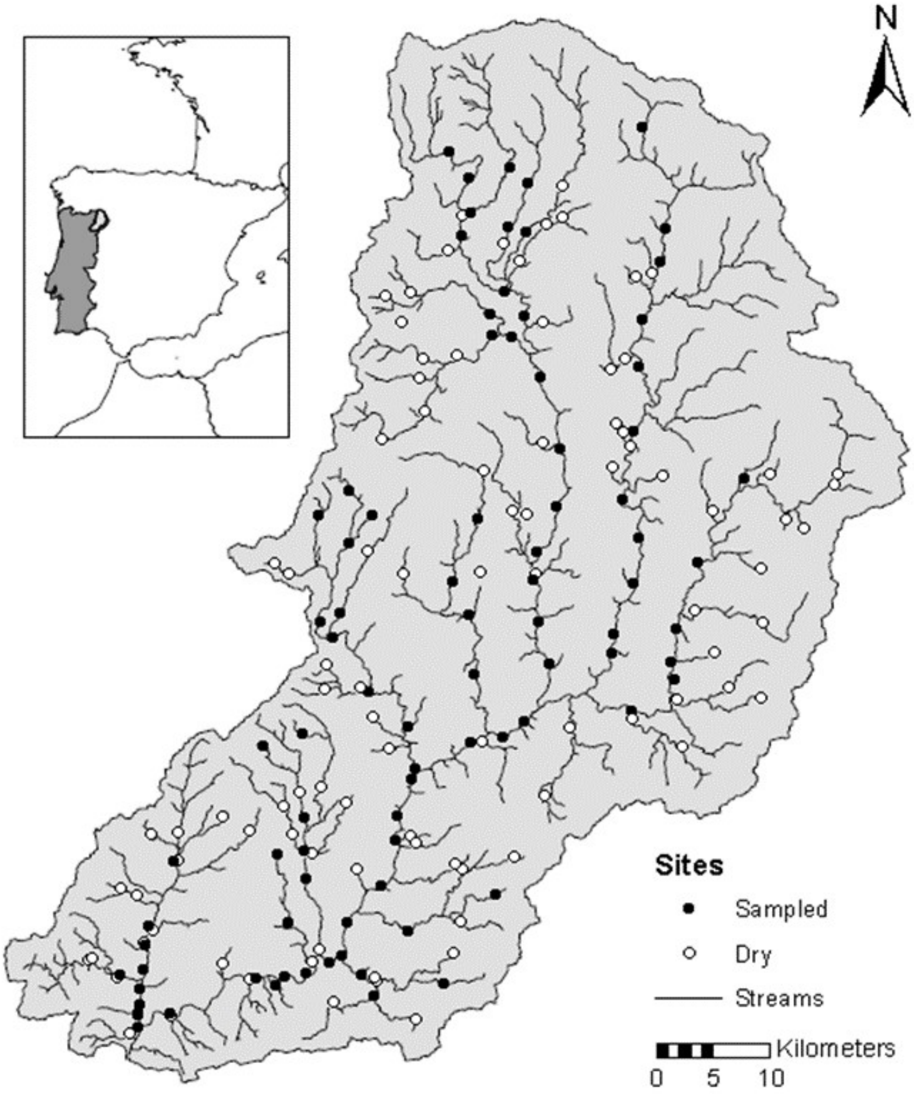

Figure S1. Map of the study area, showing the location of sites visited in the summer of 2012 (June 13 to July 15, and August 28 to September 15). Fish sampling was carried in 89 sites that had flowing water or isolated pools, while the remaining 95 sites were dry.

Table S1. **List of fish species sampled in the Sabor catchment in the summer of 2012. The percentage of sites with detections is provided for each species (frequency of detection; n=89 sites). Species used in distribution modelling (occurring in > 20 sites) are highlighted in bold type.**

Species	Frequency of detection (%)
**Native**	
Pseudochondostoma duriense	**56.2**
Squalius carolitertii	**53.9**
Luciobarbus bocagei	**52.8**
Squalius alburnoides	**33.7**
Achondrostoma arcasii	13.5
Salmo trutta	11.2
Cobitis paludica	10.1
**Exotic**	
Gobio lozanoi	**41.6**
Lepomis gibbosus	**31.5**
Gambusia holbrookii	14.6
Alburnus alburnus	4.5
Carassius auratus	1.1

Environmental covariates

Detection probabilities were modelled in relation to stream width and depth (Table S2), because electrofishing efficiency tends to be lower in larger and deeper watercourses (Reynolds, 1996). Also, these variables may strongly influence fish abundances, which in turn may positively affect detectability (MacKenzie et al., 2006; McCarthy et al., 2013). Mean reach width was estimated from measurements taken along four equally spaced transversal segments, whereas mean depth was estimated from three measurements taken in each of the four segments (Table S2).

Covariates for site occupancy included total annual precipitation, elevation, and Strahler's stream order, which have been widely reported to strongly influence the distribution of stream fish in Mediterranean regions (Magalhães et al., 2002; Filipe et al., 2004; Ferreira et al., 2007). The same covariates were used to model the probability of water presence at each site. Elevation at each sampling site was calculated from a 10-m resolution Digital Elevation Model (DEM) derived from 1:

25,000 topographic maps using ArcMap 10.0 (ESRI, 2011). Stream order was used to define stream size based on the hierarchy of tributaries, and it was extracted from the Catchment Characterization and Modelling database (CCM2), based on a 100-m resolution DEM (Vogt et al., 2007). Precipitation was extracted from WorldClim current climate predictors, which are based on the monthly mean interpolations from records collected over a 50-year period (1950-2000), with a 30 arc-seconds grid resolution (approximately 1km^2; Hijmans et al., 2005). The resolution of this variable was converted to match the 10-m resolution of the DEM.

Table S2 **Description and summary statistics (mean ± SD, range) of environmental variables used to model variation in detection rate and occupancy probability.**

Variables	Description	Mean ± SD	Range
**Detection model**			
Stream width (m)	Mean of four width measurements taken at equally spaced transversal segments along the 50-m sampling reach.	9.7±6.8	1.5-34.8
Stream depth (cm)	Mean of depth measurements taken at, 25%, 50%, and 75% of the length of each transversal segment.	34±19	9-100
**Occupancy model**			
Elevation (m)	Altitude above sea level extracted from a 10-m resolution digital elevation model derived from 1: 25,000 topographic maps	469±202	110-800
Precipitation (mm)	Average of total annual precipitation (1950-2000), extracted from WorldClim and downscaled to a 10-m resolution.	686±82	568-894
Stream order	Strahler's stream order, extracted from the River and Catchment Database CCM2	2.6±1.4	1-6

Appendix S2 Interval-censored time to detection model

The modelling procedure was based on the exponential time to detection model developed by (Garrard et al., 2008, 2013), using a modified formulation of interval-censored parametric survival models to deal with cases when detections are recorded in time intervals instead of continuously (Chen et al., 2012; Kleinbaum & Klein, 2012). Under the general parametric survival model for the time T observed for a certain event of interest (e.g. the first detection of a species), the probability of observing an event after time t (i.e. time of the event T is greater than t) equals the survival distribution for time t:

$$\Pr(T > t) = S(t) \quad \text{Eqn S1}$$

and the probability of observing an event before a time t equals the complementary probability of observing the event after time t:

$$\Pr(T \leq t) = 1 - S(t) \quad \text{Eqn S2}$$

Therefore, the probability of the event occurring in a given time interval defined by a lower bound (t_1) and a upper bound (t_2) is the probability of observing the event before the time t_2 minus the probability of observing the event before time t_1:

$$\Pr(t_1 < T \leq t_2) = 1 - S(t_2) - \left(1 - S(t_1)\right) = S(t_1) - S(t_2) \quad \text{Eqn S3}$$

This very general formulation can be parameterized using one of several available distributions of survival times, including in the simplest case the exponential model, which is fully described by a single parameter – the detection rate (λ):

$S(t) = e^{-\lambda t}$ Eqn S4

In the context of species detection, the previous equations can be combined to estimate the likelihood of species detection in a given time interval. Considering that a species occupies a site *i*, and that first detections follows an exponential distribution with detection rate λ, the likelihood of observing a first-detection event (denoted $\delta_i = 1$) during a survey interval defined by $(t_{1,i}, t_{2,i}]$, is

$$(\delta_i = 1, t_{1,i}, t_{2,i} \middle| \lambda) = e^{-\lambda t_{1,i}} - e^{-\lambda t_{2,i}}, \quad \text{Eqn S5}$$

and the likelihood of not detecting ($\delta_i = 0$) the species during a survey of duration T_i is

$$l(\delta_i = 0|\lambda, T_i) = e^{-\lambda T_i}. \quad \text{Eqn S6}$$

These equations assume that the event will occur, even if it is not detected during the survey time. However, in contrast to survival analysis, it is uncertain whether a species is present or absent, and so it may remain unrecorded either because it is absent or because it is present but remained undetected. This possibility is considered in time to detection models by including in equations S5 and S6 the probability that the species actually occupy the site. That is, the probability of detection in a given time interval under unknown occupancy is given by

$$l\left(\delta = 1, t_{1,i}, t_{2,i} \middle| \lambda, \psi\right) = \psi.\left(e^{-\lambda t_{1,i}} - e^{-\lambda t_{2,i}}\right),$$

Eqn S7

$$l(\delta_i = 0|\lambda, \psi, T_i) = \psi\left(e^{-\lambda T_i}\right) + (1 - \psi),$$

where λ is the rate at which detection events occur, ψ is the probability of the species occupying a site, T_i is the survey time at site *i*. This formulation implies that the likelihood of not recording a species during a survey ($\delta = 0$) is now a function of both imperfect detection ($\psi . e^{-\lambda T_i}$) and true absence ($1 - \psi$) (Garrard et al., 2008, 2013). The model assumes that the species is available for detection during the entire sampling period, which is a reasonable assumption, considering that all fish occurring in a stream reach are exposed to electrofishing sampling (Reynolds, 1996). However, a more general treatment of imperfect detection would have to describe both the probability of a species being available for sampling and the probability of detection given availability (e.g., Kéry & Schmidt, 2008), but this was beyond the scope of this study.

In the above we have specified the observation process as the exponential model from eqn S4 onward; returning now to full generality we give the likelihood expressions for any given parametric detection-time distribution $S(\mathrm{t}) = \mathrm{S}(\mathrm{t}, \theta)$ with vector of parameters θ:

$$\begin{aligned} l\left(\delta_i = 1, t_{1,i}, t_{2,i} \mid \theta, \psi\right) &= \psi\left(S(t_{1,i}, \theta) - S(t_{2,i}, \theta)\right), \\ l\left(\delta_i = 0 \mid \theta, \psi, \mathrm{T}_i\right) &= \psi\ S(\mathrm{T}_i, \theta) + \left(1 - \psi\right). \end{aligned} \qquad \text{eqn S8}$$

Whereas the exponential model has the property that the detection probability for any time interval $(t_1, t_2]$ depends only on the length $t_1 - t_2$ of the interval (the memoryless property, Murphy et al., 2002), more general distributions such as the 2-parameter Weibull distribution allow detection probabilities for equal intervals to increase or decrease with later times. This allows, for example, for inclusion of changing detection probability over time due for instance to disturbances from survey efforts.

Due to this property, when using the exponential model, i.e. the simplest of parametric survival models (Kleinbaum & Klein, 2012), the interval-censored time to detection model with equal intervals is the mathematical equivalent of the occupation-detection models with removal design (MacKenzie et al., 2006) with equal detection probabilities for each of a series of discrete surveys. In this approach researchers record species detections at the end of intervals of length Δt, stopping after the 1st detection or once a predefined maximum number (K) of intervals has elapsed. If time to first detection of species follows an exponential survival time model with detection rate λ then the detection probability in each interval is $\mathrm{p} = 1 - \mathrm{e}^{-\lambda\Delta\mathrm{t}}$.

When the first detection at a site happens within the interval defined by times t_1 and t_2, and calling that interval the k_i-th repeat visit we can developed the likelihood in Eqn. S7 as follows:

$$l(t_1, t_2|\lambda, \psi) = \psi.\left(e^{-\lambda t_1} - e^{-\lambda t_2}\right) = \psi.\left[\left(e^{-\lambda}\right)^{t_1} - \left(e^{-\lambda}\right)^{t_2}\right] = \psi.\left[(1 - \mathrm{p})^{t_1/\Delta t} - (1 - \mathrm{p})^{t_2/\Delta t}\right]$$

If we keep Δt constant then by definition of the k_i-th repeat visit, we know that $t_2/\Delta\mathrm{t} = k_i$ and $t_1/\Delta\mathrm{t} = k_i - 1$, so we have:

$$l(t_1, t_2|\lambda, \psi) = \psi.\left[(1 - \mathrm{p})^{k_i - 1} - (1 - \mathrm{p})^{k_i}\right] = \psi.(1 - \mathrm{p})^{k_i - 1}.(1 - 1 + p) = \psi.(1 - \mathrm{p})^{k_i - 1}.p$$

which is the expression for removal sampling detection data for a site where 1st detection happens at the k_i-th repeat visit, i.e. we get (k_i − 1) non-detections followed by the single detection (MacKenzie et al., 2006).

When there are no detections after a total survey time T_i (i.e. K repeat visits with K = T/Δt):

$$l(T_i|\lambda, \psi) = \psi\left(e^{-\lambda T_i}\right) + (1 - \psi) = \psi(1 - \mathrm{p})^K + (1 - \psi)$$

The first-detection time models considered here generalize the above scheme to allow (i) uneven sampling times/intervals, (ii) variation of detection rates between sites and (iii) variation of detection rates as a function of time by employing non-exponential parametric survival models (e.g. Weibull model Kleinbaum & Klein, 2012).

Appendix S3 Code used to fit the time to detection model using WinBUGS

```
model {
# priors
a0 ~ dnorm(0, .1) I(-10, 10) # Intercept for Water availability
b0 ~ dnorm(0, .1) I(-10, 10) # Intercept for occupation
g0 ~ dnorm(0, .1) I(-10, 10) # Intercept for detection

for (n in 1:Xocc) { # mean effects in occupation
	a[n] ~ dnorm(0, .1) I(-10, 10) #Effects Water availability
	b[n] ~ dnorm(0, .1) I(-10, 10) #Effects occupancy
}

for (m in 1:Xdet) {
	g[m]~ dnorm(0, .1) I(-10, 10) #Effects Detection
}

a.sp ~ dnorm(0, .1) I(-10, 10) #Spatial Effect on Water availability
b.sp ~ dnorm(0, .1) I(-10, 10) #Spatial Effect on occupation

#Spatial Autologistic term computation

for (i in 1:nsite) {
	for(j in 1:nnb[i]) {
		autoZ[i,j] <- Z[nblists[i,j]]
	}
	Z.sp[i]<-inprod(autoW[i,1:nnb[i]],autoZ[i,1:nnb[i]])
}

#Model
for (i in 1:nsite) {

	#Water availability
	lW[i] <- a0 + inprod(a[ ], X1[i, ]) + a.sp * W.sp[i]
	pW[i] <- 1/(1+exp(-lW[i]))
	W[i] ~ dbern(pW[i])

	#Occupancy Model
	lpsi[i] <- b0 + inprod(b[ ], X1[i, ]) - (1 - W[i]) * pow(10, 9) + b.sp * Z.sp[i]
	psi[i] <- 1/(1+exp(-lpsi[i]))

	Z[i] ~ dbern(psi[i]) #True state occupation

	#Detection rate
	lambda[i] <- exp(g0 + inprod(g[], X2[i,]))

	#Survival function for Left bound
	S1[i] <- exp(-lambda[i] * y1[i])

	#Survival function for Right bound
```

```
        S2[i] <- exp(-lambda[i]   * y2[i])

        #when species is detected
        pp[i] <- (S1[i] - S2[i]) * psi[i]

        #when species is not detected
        pn[i] <- S2[i]  * psi[i] + (1 - psi[i])

        #Select likelihood
        p[i] <- (d[i]*pp[i] + (1-d[i])*pn[i]) #d[i] = 1 where detected, d[i]= 0 where not detected

        ones[i] ~ dbern(p[i]) #ones trick

        # Probability of detecting an individual at site i (Evaluation purposes)
        p1[i] <- psi[i] * (1 - exp(-lambda[i] * TT[i]))
        Res[i] <- d[i] - p1[i] #Residuals

        #Replicate observations
        d_rep[i] ~ dbern(p1[i]) #Generate replicate observations
        Res_rep[i] <- d_rep[i] - p1[i] #Replicate residuals
}

fit <- sum(Res[])             # Sum of residuals for actual data set
fit.new <- sum(Res_rep[])      # Sum of residuals for new data set
test <- step(fit.new - fit)                 # Test whether new data set more extreme
bpvalue <- mean(test)                       # Bayesian p-value

# Catchment area extrapolation

#Spatial Autologistic term computation
for (i in 1:nsite2) {
        for(j in 1:nnb2[i]) {
                autoZ2[i,j] <- Z[nblists2[i,j]]
        }
        Z2.sp[i]<-inprod(autoW2[i,1:nnb2[i]],autoZ2[i,1:nnb2[i]])
}


for (j in 1:nsite2) {
        #Water availability
        lW2[j] <- a0 + inprod(a[ ], X3[j, ]) + a.sp * W.sp2[j]
        pW2[j] <- 1/(1+exp(-lW2[j]))
        W2[j] ~ dbern(pW2[j])

        #Occupancy Model
        lpsi2[j] <- b0 + inprod(b[ ], X3[j, ]) - (1 – W2[j]) * pow(10, 9) + b.sp * Z.2sp[j]
        psi[j] <- 1/(1+exp(-lpsi2[j]))
}
}# end of model
```

Appendix S4 Response curves to environmental variables

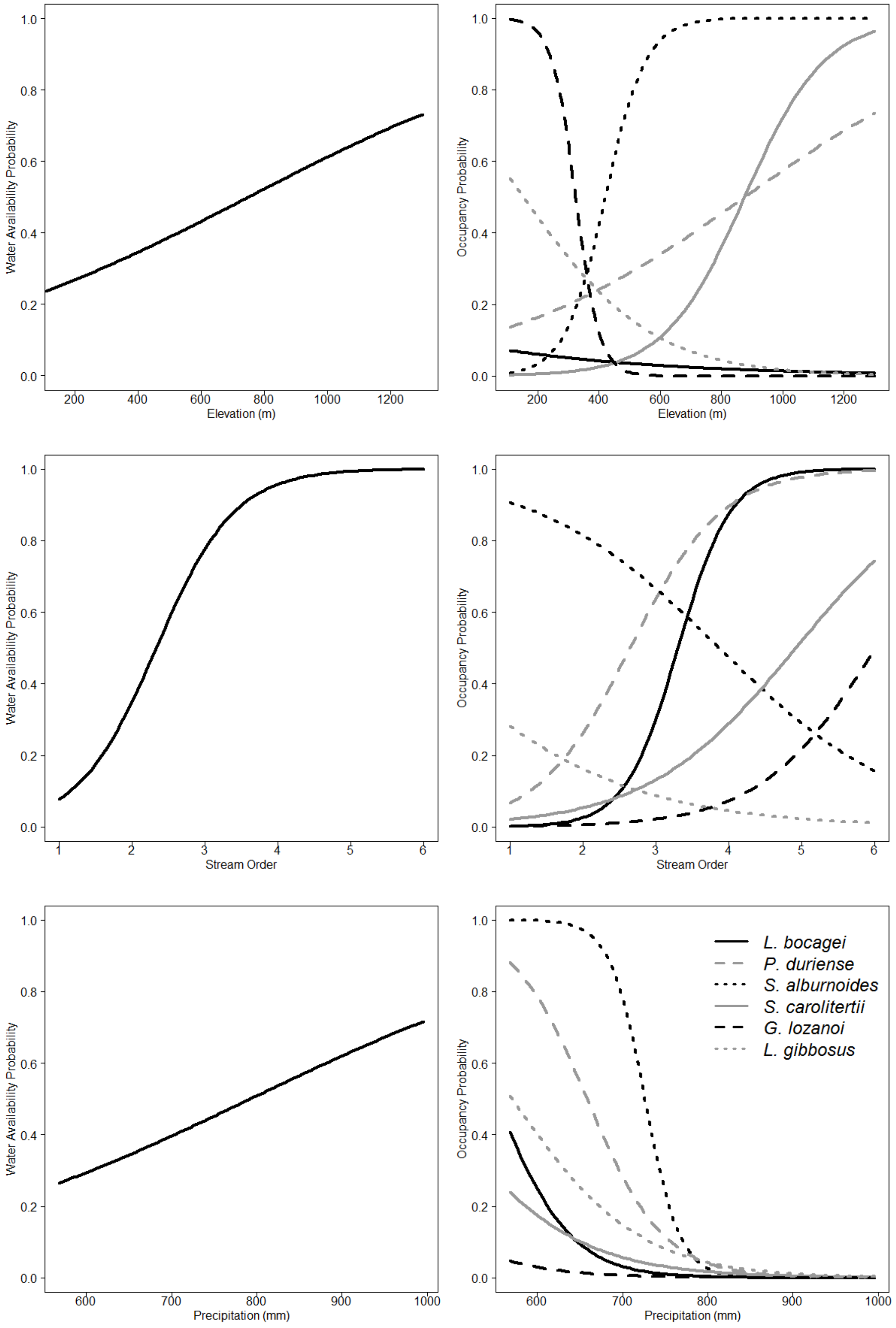

Figure S2 Relationships estimated from hierarchical occupancy-detection models between environmental variables and both the probability of the watercourse having water during the sampling visit, and the probability of occupancy for each fish species when surface water is present.

Appendix S5 Maps of prediction uncertainty

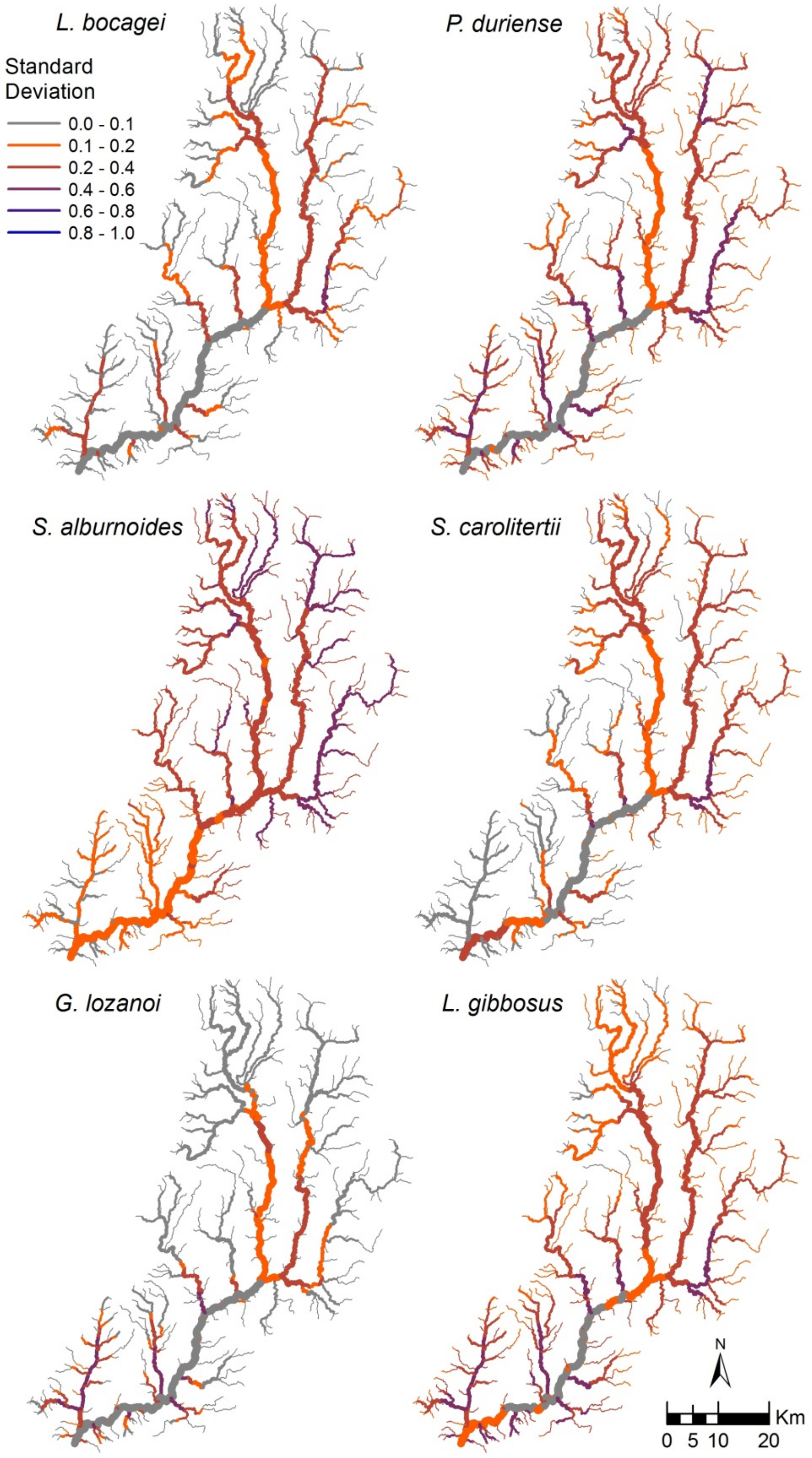

Figure S3 Maps of model prediction uncertainty for predicted distributions of six stream fish species in the Sabor catchment. Uncertainty was estimated from the standard deviation of the posterior distribution of occupancy probabilities derived from the best-supported model for each species.

Appendix S6 Supplementary references

Chen D.-G., Sun J., & Peace K. (2012) *Interval-Censored Time-to-Event Data: Methods and Applications.* Chapman and Hall/CRC,

ESRI (2011) ArcMap Release 10.0.

Ferreira M.T., Sousa L., Santos J.M., Reino L., Oliveira J., Almeida P.R., & Cortes R. V. (2007) Regional and local environmental correlates of native Iberian fish fauna. *Ecology of Freshwater Fish*, **16**, 504–514.

Filipe A.F., Marques T.A., Tiago P., Ribeiro F., Da Costa L.M., Cowx I.G., & Collares-Pereira M.J. (2004) Selection of Priority Areas for Fish Conservation in Guadiana River Basin, Iberian Peninsula. *Conservation Biology*, **18**, 189–200.

Garrard G.E., Bekessy S.A., McCarthy M.A., & Wintle B.A. (2008) When have we looked hard enough? A novel method for setting minimum survey effort protocols for flora surveys. *Austral Ecology*, **33**, 986–998.

Garrard G.E., Mccarthy M. a., Williams N.S.G., Bekessy S. a., & Wintle B. a. (2013) A general model of detectability using species traits. *Methods in Ecology and Evolution*, **4**, 45–52.

Hijmans R.J., Cameron S.E., Parra J.L., Jones P.G., & Jarvis A. (2005) Very high resolution interpolated climate surfaces for global land areas. *International Journal of Climatology*, **25**, 1965–1978.

Kleinbaum D.G. & Klein M. (2012) *Survival Analysis.* Springer New York, New York, NY.

MacKenzie D.I., Nichols J.D., Royle J.A., Pollock K.H., Bailey L.L., & Hines J.E. (2006) *Occupancy Estimation and Modeling: Inferring Patterns and Dynamics of Species Occurrence.* Elsevier Academic Press, USA.

Magalhães M.F., Beja P., Canas C., & Collares-Pereira M.J. (2002) Functional heterogeneity of dry-season fish refugia across a Mediterranean catchment: the role of habitat and predation. *Freshwater Biology*, **47**, 1919–1934.

Magalhães M.F., Beja P., Schlosser I.J., & Collares-Pereira M.J. (2007) Effects of multi-year droughts on

fish assemblages of seasonally drying Mediterranean streams. *Freshwater Biology*, **52**, 1494–1510.

McCarthy M. a., Moore J.L., Morris W.K., Parris K.M., Garrard G.E., Vesk P. a., Rumpff L., Giljohann K.M., Camac J.S., Bau S.S., Friend T., Harrison B., & Yue B. (2013) The influence of abundance on detectability. *Oikos*, **122**, 717–726.

Murphy K.E., Carter C.M., & Brown S.O. (2002) The exponential distribution: the good, the bad and the ugly. A practical guide to its implementation. *Annual Reliability and Maintainability Symposium. 2002 Proceedings*, 550–555.

Penczak T. & Głowacki Ł.B. (2008) Evaluation of electrofishing efficiency in a stream under natural and regulated conditions. *Aquatic Living Resources*, **21**, 329–337.

Podani J. & Csontos P. (2006) Quadrat size dependence, spatial autocorrelation and the classification of community data. *Community Ecology*, **7**, 117–127.

Reynolds J.B. (1996) *Electrofishing. Fisheries Techniques.* American Fisheries Society, Bethesda, Maryland.

Vittinghoff E. & McCulloch C.E. (2007) Relaxing the rule of ten events per variable in logistic and Cox regression. *American journal of epidemiology*, **165**, 710–8.

Vogt J. V, Soille P., Jager A. De, Rimavičiūtė E., Mehl W., Haastrup P., Paracchini M.M.L., Dusart J., Bódis K., Foisneau S., & Bamps C. (2007) Developing a pan-European Database of Drainage Networks and Catchment Boundaries from a 100 Metre DEM. *Proceedings 10th AGILE Int. Conference on Geographic Information Science*, 8–11.

Zalewsky M. & Cowx I.G. (1990) Factors affecting the efficiency of electric fishing. *Fishing with electricity: applications in freshwater fisheries management* pp. 89–111. Fishing News Books, Oxford, UK.
